# SOCIOCULTURAL DETERMINANTS IN PAIN PERCEPTION AND MANAGEMENT AMONG OLDER CHINESE AMERICANS

**DOI:** 10.1093/geroni/igz038.2619

**Published:** 2019-11-08

**Authors:** Simona Kwon, Jazmine Wong, Janet Pan, Andrew Rosenberg, Germaine Cuff, Myint Aye

**Affiliations:** 1 NYU School of Medicine, New York, New York, United States; 2 NYU Langone Hospital Brooklyn, Brooklyn, New York, United States

## Abstract

Background: Chinese Americans make up the largest Asian American subgroup in the US. Data from a large health system indicate that older Chinese Americans experience lower satisfaction in pain management after surgery compared to all other racial/ethnic groups. Objective: To understand pain experience among older Chinese American patients to improve pain satisfaction strategies Methods: A mixed methods study was conducted, including: 1. A scoping review of the peer-reviewed published literature; 2) face-to-face survey; and 3) qualitative interviews. 14 Chinese American postsurgical patients >65 years of age were recruited for the survey and interview with a trained bilingual Community Health Worker. Questions from the Survey on Disparities in Quality of Healthcare and Kleinman’s Explanatory Model of Illness guided the data collection tools. Results: The 31 studies identified in the review were largely observational; none assessed pain control or management interventions for older Chinese Americans. Most participants reported experiencing a language barrier that hindered healthcare staff communication during hospital stay. Even with an interpreter, limited English proficient patients reported lower understanding of health information compared to those who did not need interpretation. Ideas of “pushing through” pain, perceiving physicians as “busy people,” and mismatch in pain assessment tools contributed to pain attendance delay. Facilitators to care included family support, culturally and linguistically-tailored tools, and availability of cultural remedies. Conclusions: This mixed-methods study identified key themes including socio-cultural barriers and facilitators to effective pain care and management. Findings will inform tools and resources to better capture and address pain management in Chinese Americans.

